# Two Sudden and Unexpected Deaths of Patients with Schizophrenia Associated with Intramuscular Injections of Antipsychotics and Practice Guidelines to Limit the Use of High Doses of Intramuscular Antipsychotics

**DOI:** 10.1155/2016/9406813

**Published:** 2016-08-15

**Authors:** Nasratullah Wahidi, Katie M. Johnson, Allen Brenzel, Jose de Leon

**Affiliations:** ^1^Department of Psychiatry, College of Medicine, University of Kentucky, Lexington, KY 40509, USA; ^2^Department of Behavioral Health, Developmental and Intellectual Disabilities, Frankfort, KY 40621, USA; ^3^Eastern State Hospital, University of Kentucky Mental Health Research Center, Lexington, KY 40511, USA; ^4^Psychiatry and Neurosciences Research Group (CTS-549), Institute of Neurosciences, University of Granada, 18971 Granada, Spain; ^5^Biomedical Research Centre in Mental Health Net (CIBERSAM), Santiago Apóstol Hospital, University of the Basque Country, 01004 Vitoria, Spain

## Abstract

Intravenous haloperidol has been associated with torsades de pointes (TdP). These two sudden deaths were probable adverse drug reactions (ADRs) following intramuscular (IM) antipsychotics. The autopsies described lack of heart pathology and were highly compatible with the possibility of TdP in the absence of risk factors other than the accumulation of antipsychotics with a high serum peak after the last injection, leading to death within hours. The first case was a 27-year-old African-American male with schizophrenia but no medical issues. His death was probably caused by repeated IM haloperidol injections of 10 mg (totaling 35 mg in 2 days). The second case involves a 42-year-old African-American female with metabolic syndrome. Her probable cause of death was the last ziprasidone IM injection of 20 mg in addition to (1) three extra haloperidol doses (2 hours before the ziprasidone injection, 5 mg oral haloperidol; approximately 21 hours earlier, 5 mg oral haloperidol; and 2 days prior, one 10 mg IM haloperidol injection), (2) 10 mg/day of scheduled oral haloperidol for 6 days before death, and (3) a long-acting paliperidone injection of 156 mg 18 days before death. The study of haloperidol glucuronidation and its impairment in some African-Americans is urgently recommended.

## 1. Introduction

Thioridazine was introduced in the US market in 1959 [1]. In 1964 [2], Kelly et al. reported that thioridazine was associated with quinidine-like electrocardiogram (EKG) abnormalities in 28 patients and with two cases of ventricular tachycardia leading to death in patients taking 1500 mg/day and 3600 mg/day, respectively. The awareness of these deaths before publication led the pharmaceutical company to talk to Ban and St. Jean [3]. Ban proposed completing a prospective EKG study using four doses of thioridazine and four doses of chlorpromazine and trifluoperazine as controls. EKG abnormalities resembling those caused by quinidine and hypokalemia were present in all 6 patients taking thioridazine (versus 3/6 for chlorpromazine and 1/6 for trifluoperazine) [3]. Before Ban and St. Jean published their study, Desautels et al. identified a patient who survived ventricular tachycardia while receiving 1500 mg/day of thioridazine for 6 weeks [4]. Some other studies were published regarding the matter [1], including one by Simpson as senior author [5] describing thioridazine-induced EKG changes as very likely in the elderly and, therefore, recommending against thioridazine as a drug for the elderly. According to Shorter, Ban tried to convince the pharmaceutical company of the clinical relevance of thioridazine-induced arrhythmias but was not successful [1]. As a matter of fact, the company did not change the prescribing information to warn physicians of the risk of thioridazine causing torsades de pointes (TdP) until 2000 [1]. Finally, in 2006, more than 40 years after the first deaths associated with thioridazine, the drug was withdrawn from the US market [6].

After slowly increasing awareness, the relationship between drugs, including antipsychotics, and TdP, a potentially lethal adverse drug reaction (ADR), became irrefutable in the 1990s and the US Food and Drug Administration (FDA) had no choice but to intervene. The turning point came in 1996 [7]. Terfenadine was a second-generation antihistamine that had been approved in the USA in 1985 [6] after no systematic studies on drug-drug interactions (DDIs) and on drug metabolism. The randomized controlled trials (RCTs) in healthy subjects with limited comedication had demonstrated that it was a very safe drug. Once approved, terfenadine was widely used in the general population including in patients who were taking erythromycin, ketoconazole, and itraconazole. These 3 drugs are powerful cytochrome P450 (CYP) 3A4 (CYP3A4) inhibitors and terfenadine was metabolized by CYP3A4. In 1996, the FDA became aware [7] that there were at least 125 deaths in the US caused by terfenadine. Patients taking any of these major CYP3A4 inhibitors had major accumulations of terfenadine with very high serum concentrations leading to TdP. The FDA required warning labels in the prescribing information for terfenadine and the CYP3A4 inhibitors; terfenadine was finally withdrawn from the market in 1998 [6]. The terfenadine deaths explained by DDIs led to the FDA's awareness of the need to study CYP metabolism and DDIs for new drug submissions. The requirements were progressively increased in the late 1990s, after several other drugs metabolized by CYP3A4 and with potential to cause TdP were withdrawn from the market [6].

Sertindole, a second-generation antipsychotic, was introduced in Europe in 1996 [8]. It rapidly became associated with sudden cardiac deaths and an in vitro study demonstrated that sertindole has high affinity as an antagonist of the cardiac potassium channel [9]. In humans, the delayed rectifier potassium current is mediated by the ion channel KCNH2 encoded by the human ether-a-go-go–related gene (HERG) [9]. After the publication of this article describing that sertindole was an antagonist of the channel encoded by HERG [9], it became evident that first-generation antipsychotics, such as thioridazine, must have the same antagonist properties at the same cardiac channel; this also explained why hypokalemia is a risk factor for antipsychotic-induced TdP [10].

Since TdP is a very rare event, in the late 1990s the FDA proposed using prolongation of the QT interval as a TdP risk marker. This was a complex decision since there is general agreement that the QT interval needs to be corrected by heart frequency, which is called corrected QT (QTc), but there are several formulas for doing it and no agreement among experts concerning which one is best. Another issue is how good QTc prolongation proves to be as a sign of the blockade of the cardiac potassium channel, which is determined by drug affinity and the serum concentrations in a specific patient. Thioridazine appears to have the highest affinity among antipsychotics and the use of very high doses >1,000 mg/day, such as the doses used in the 1960s, led to very high serum concentrations and extremely high potential for TdP in many, if not all, patients.

In the late 1990s, the company developing ziprasidone was trying to introduce it into the US market, but ziprasidone has affinity for the human cardiac potassium channels and caused prolongations of the QTc interval. To allay the FDA's concerns, ziprasidone's marketer decided to complete a milestone prospective randomized study [11] comparing 6 oral antipsychotics (haloperidol, quetiapine, olanzapine, risperidone, thioridazine, and ziprasidone) by themselves and in the presence of CYP inhibitors (fluvoxamine for CYP1A2, ketoconazole for CYP3A4, and paroxetine for CYP2D6). With the specific doses used in that study, thioridazine was associated with greater QTc prolongations with a mean increase of 30.1 milliseconds (ms), ziprasidone was second with 15.9 ms, and haloperidol was third with 7.1 ms. Following this study, ziprasidone was permitted in the US market in 2001 with some warnings about QTc prolongation in the prescribing information. Then, some of the first-generation antipsychotics started to be withdrawn from some markets due to TdP risk. This led to major discussions in the US and international journals [12–14] and major disagreement on the advertising by the company marketing antipsychotics about the clinical relevance of antipsychotic-induced QTc prolongations and the virtues or weaknesses of various second-generation antipsychotics regarding their effects on the QTc interval. The problems [14] are that (1) sudden death on oral antipsychotics has a very low incidence: around 1 in every 10,000 patients. This requires 10,000 patients taking medications for an extended period to identify a few cases, making it very difficult to study; and (2) these large pharmacoepidemiological naturalistic studies include sudden death cases with multiple confounding factors and no guarantee that they are explained by TdP versus other causes of sudden death or by TdP explained by the combination of multiple factors besides an antipsychotic. Review articles started recommending a QTc limit (e.g., >500 ms) as a risk marker for TdP [12]. This became controversial since other authors recommended other values, such as >450 ms [13]. Moreover, the literature clearly indicated that women have a greater mean QTc; therefore, a sex-corrected QTc limit for predicting risk for TdP may be needed (e.g., >450 ms in men and >470 ms in women) [13].

These controversies concerning QTc prolongation are not easy to resolve since there is limited reliable data on TdP cases caused by antipsychotics. Summarizing our current knowledge [14], we can describe most cases of drug-induced TdP as occurring in the context of substantial prolongation of the QTc interval, typically to values >500 ms, but QTc alone is a relatively poor predictor of arrhythmic risk in any individual patient. Some drugs that substantially prolong the QTc interval produce very low rates of TdP while others have much smaller QTc effects but are considerably more prone to cause TdP [15]. The psychiatric literature describing clinical cases of TdP is very complex [14], since the cases are frequently associated with polypharmacy and DDIs, with a pharmacodynamic component involving multiple drugs with HERG channel inhibitory properties and sometimes a pharmacokinetic component, an inhibitor increasing the plasma concentrations of one or several of the drugs. Other risk factors for TdP such as female gender, bradycardia, hypokalemia, and hypomagnesemia may be important, too [16].

Since 2001, oral ziprasidone has occasionally been associated with TdP, but most of the cases include patients with other TdP risk factors [17]. A large 1-year mortality study randomly assigning its 18,000 patients to ziprasidone or olanzapine concluded that these drugs have similar nonsuicide mortality, but the study acknowledged that it did not have enough power to detect rare events such as TdP [18]. In a comprehensive review of the literature, sponsored by ziprasidone's marketer and focused on the company's RCT and the postmarketing surveillance data, Camm et al. [19] concluded that ziprasidone is safe if used as indicated. On the other hand, a more recent pharmacoepidemiology review by independent investigators suggested that there were 3 antipsychotics definitively associated with TdP: amisulpride, haloperidol, and ziprasidone [20]. As population clinical data [19] and laboratory pharmacological studies [21] indicate that drug-induced QTc prolongations are without doubt driven by higher serum concentrations, the parenteral administration of any of these antipsychotics, which is associated with much higher peak concentrations than oral administration, should substantially increase the risk of antipsychotic-induced TdP in patients receiving intramuscular (IM) formulations. IM ziprasidone was approved in the US for agitation in schizophrenia patients in 2002. After a healthy young Chinese male patient with schizophrenia demonstrated a QTc interval prolongation of 83 ms after receiving a single IM injection with 20 mg of ziprasidone, Li et al. [22] completed a systematic review on the effects of IM ziprasidone on the QTc interval prolongation. They identified 19 trials in English or Chinese with a total of 1428 patients, many using haloperidol IM as a control. Their review identified two cases of patients who experienced symptoms probably related to QTc prolongation after IM ziprasidone. If one assumes that published IM ziprasidone cases are representative, one can estimate you need only 1,000 patients on IM ziprasidone to identify symptoms of TdP versus 10,000s required to identify sudden deaths associated with oral antipsychotics. In the systematic review, mean QTc change from baseline to end of each trial ranged from −3.7 to 12.8 ms after IM ziprasidone (compared with −3.5 to 14.7 ms on haloperidol IM). Four RCTs were used to calculate a meta-analysis of QTc interval prolongation; it showed no significant differences between IM ziprasidone and IM haloperidol groups. In summary, IM ziprasidone appeared to have some risk for TdP that did not look different from IM haloperidol.

The history of haloperidol-induced TdP is also as protracted as that of thioridazine-induced TdP. Haloperidol is a first-generation antipsychotic marketed in the 1960s but with the peculiarity of having formulations for IM and intravenous (IV) administration that are more risky for TdP since they provide much higher peak serum concentrations than oral formulations. IV haloperidol is mainly used for sedation by internists rather than by psychiatrists and internists use it in continuous administration controlled by nurses; there is potential for huge doses since haloperidol is not a potent drug for sedating patients. Psychiatrists tend to use haloperidol IM and combine it with other more sedating drugs (e.g., lorazepam and/or diphenhydramine). During the 1990s and 2000s, there was a progressive increase in cases demonstrating the association between extremely high accumulated doses of haloperidol IV and TdP [23–26], to the point that the FDA required a warning in the US haloperidol prescribing information in 2007 [27]. Based on the case reports of potentially fatal cardiac events, the FDA warned that haloperidol is not approved for IV administration, but if IV administration is used, EKG monitoring should be performed. Meyer-Masseti et al. [27] considered the FDA's recommendations in 2007 confusing. The FDA warned that QTc prolongation and risk of TdP were increased with IV administration of haloperidol or administration of haloperidol at greater-than-recommended doses in any formulation. However, Meyer-Masseti et al. [27] stressed that neither the “typical” dosing range nor the minimum dose associated with these cardiac ADRs was specified in the haloperidol prescribing information. In their review of the literature [27], Meyer-Masseti et al. identified a total of 70 cases of IV haloperidol associated with QTc prolongation and/or TdP. When postevent QTc data were reported, QTc was prolonged >450 ms in 96% of the cases. Most (97%) of the patients had additional risk factors for TdP, mainly the coprescription of other proarrhythmic agents. Patients experiencing haloperidol-associated TdP received a wide range of cumulative doses from 5 mg to 645 mg. In their review, Meyer-Masseti et al. do not address the crucial issue: what do we know about TdP only caused by IV haloperidol in the absence of other major risk factors? It is obvious that IV haloperidol should not be used in patients with TdP risk factors, but we do not know what doses of IV haloperidol may be risky in patients with no known TdP risk factors.

The association between oral and IM haloperidol with TdP has received much less attention in the literature than the association with IV haloperidol. Jackson et al. [28] published a TdP case associated with 4 mg oral haloperidol (2 doses of 2 mg separated by 8 hours) in a 66-year-old woman. Harvey et al. [29] studied 12 volunteers with schizophrenia who were given a single IM injection of 7.5 mg haloperidol or 4 mg lorazepam in a blinded, randomized, placebo-controlled crossover design. Mean changes in the QTc interval in those receiving the haloperidol IM dose ranged from 3.6 to 5.1 ms, depending on the formula used to correct for heart frequency. Harvey et al. [29] concluded that, on average, this dose of IM haloperidol led to minimal prolongation of the QT interval and this effect was of theoretical concern in individuals with risk factors for TdP but seemed unlikely to be a problem in the vast majority of patients. In our literature searches in PubMed, we have not been able to identify clinical studies of QTc prolongation using IM haloperidol formulations with repeated administration, such as those used by psychiatrists to control agitation in the clinical environment or case reports of TdP associated with IM haloperidol. There are IM ziprasidone RCTs using haloperidol IM as a control, but most of the published articles focused on the mean increases in QT prolongation rather than the extreme cases and frequently considered haloperidol-induced QTc changes as not clinically relevant (reviewed in Table 1 of [22]). 

In this paper, we present two cases of sudden death after IM injections of antipsychotics (haloperidol in the first case and a haloperidol-ziprasidone combination in the second case) with autopsies indicating lack of heart pathology and high compatibility with the possibility of TdP. After using two different ADR scales in each patient [30, 31], the authors consider these two sudden deaths to be probable ADRs.

## 2. The Patient Sample That Led to Two Cases

The state of Kentucky is located in the center of the United States and has a population of approximately 4 million people. Most Kentuckians are Caucasians; less than 10% are African-Americans and small numbers of people are from other racial/ethnic backgrounds. The majority of adult patients with severe mental illnesses (SMIs) get admitted to 4 state psychiatric hospitals and 1 forensic facility. According to our centralized database with admissions data, in the last 15 years the annual number of different patients admitted to these four psychiatric state hospitals has ranged between 5,556 and 7,106. Our experience with large published studies [32, 33] in these admitted populations indicates that there are three main groups of diagnoses justifying admission, each approximating one-third of the cases: (1) severe mood disorders, (2) schizophrenia and related psychoses, and (3) complications of substance use disorders [32, 33].

Since 2002, a state mortality review process has provided outside review of the deaths at any state facility, in addition to the internal regulatory process at each facility. The facilities include not only these state psychiatric hospitals but also 3 nursing homes and 4 long-term care facilities for adults with intellectual disabilities. Since 2008, some community deaths have been reviewed as well. Focusing only on deaths occurring during admission at state psychiatric hospitals over 14 years (from 2002 to January 2016), we identified 95 deaths. These 95 patients were 63% male and 87% Caucasian (11% African-American), which roughly corresponds to the admission demographics in our state psychiatric hospitals, based on our published studies including thousands of patients [32, 33]. The mean age of the 95 deceased patients was 58.0 years (standard deviation 16.7 years), apparently higher than the average age of admitted patients every year, which tends to be in the late 30s [32, 33]. Obviously, older patients are more prone to die. Of these 95 deaths, two (approximately 2%) appeared highly compatible with TdP associated with antipsychotic treatment. Both patients were prescribed high doses of IM antipsychotic injections and had autopsies showing no heart abnormalities.

According to the committee's evaluation, besides these 2 deaths compatible with TdP, there were 32 other unexpected sudden deaths that may need to be considered in differential diagnosis. Only 7 of these 32 deaths had autopsies but the committee concluded that (1) 17 appear compatible with myocardial infarcts, (2) 9 appear compatible with pulmonary thromboembolism, (3) two appear compatible with sudden death in the context of epilepsy, (4) one appears compatible with a rupture of aortic aneurysm, and (5) three were of unknown origin (including one in which the request for autopsy was denied by the coroner). An antipsychotic-induced arrhythmia was not suspected in any of these 32 deaths since they were not temporally associated with recent IM injections or extremely high doses of oral antipsychotics, but there is no way of ruling out TdP with 100% certainty. An autopsy providing another cause of death (e.g., myocardial infarct) cannot rule out the possibility that the patient had an antipsychotic-induced TdP on top of a myocardial infarct. Nonetheless, the two cases described in detail in this paper were judged to be highly compatible with sudden death after the high doses of IM antipsychotics in the absence of other known risk factors for TdP.

## 3. Case Presentations

### 3.1. Case  1

#### 3.1.1. Prior History

This patient was a 27-year-old African-American male. His weight was 88 Kg (194 pounds). He had been diagnosed with chronic schizophrenia since age 22. He also had a history of polysubstance abuse and was a smoker. He had 9 prior state hospital admissions. During two of these admissions, he received one injection of 5 mg haloperidol IM to control psychosis and agitation, each without any obvious problem. During the 8th admission, he intermittently refused oral antipsychotic treatment medication that was court-ordered, which led to 2 injections of 10 mg haloperidol (and diphenhydramine 50 mg) without any problem, but the second injection was administered 3 days later, when the peak concentrations from the first injection were long gone. During the 9th admission, when he was 25 years old, an EKG showed “sinus bradycardia” (frequency of 58 beats per minute). The QT interval was read as 376 ms and the QTc as 373 ms. At that time he was taking, as oral medications, risperidone 4 mg/day, benztropine 2 mg/day, and divalproex sodium 1500 mg/day and 3 days before the EKG, the patient had also received an injection of long-acting risperidone of 37.5 mg but no other antipsychotic injections in the prior week. Three days before the EKG, electrolytes including potassium were normal. In summary, this EKG during reasonable doses of oral and long-acting risperidone demonstrated a completely normal QTc two years before the patient's death.

#### 3.1.2. Last Admission

The patient's 10th admission to state facilities lasted less than 3 days due to his death in the early morning of the third day. It was an involuntary admission with forced medication approved by the court. The patient was transferred from jail where he had had no access to tobacco smoking for weeks. Upon arrival, he was uncooperative and psychotic. His oral medications, risperidone 6 mg/day (3 mg twice a day) and benztropine 2 mg/day (1 mg twice a day), were reordered since it appeared that he had not been taking them in jail. A court-approved forced intramuscular (IM) order of haloperidol 10 mg and diphenhydramine 50 mg was to be administered if he refused any dose of oral risperidone. The admitting psychiatrist was questioned about this high haloperidol IM dose after the patient's death and provided the explanation that 3 years prior, at the same facility (his 8th admission to a state facility), the patient had received the same IM dosage of haloperidol 10 mg IM and diphenhydramine 50 mg without problems when refusing court-ordered oral medications. The psychiatrist was not aware that only 2 such injections were given during that prior admission and they were separated by 3 days. At admission the patient was uncooperative, refused a physical exam, and only cooperated with the measure of blood pressure (130/90 mm of Hg) and pulse (106 beats per minute). During the rest of the admission, he refused offers of completing the physical exam, vital signs and laboratory tests, but the available information indicated no obvious medical problems and he was essentially a medically healthy young man with no history of any serious medical problems during his 9 prior admissions to our facilities.

During the first 2 days of this admission, the patient refused all oral medications and received forced IM medication twice a day with total accumulated dosages of IM haloperidol 35 mg and diphenhydramine 200 mg. The patient received 4 IM haloperidol injections but one of the nurses made a mistake, giving only 5 mg. Therefore, the patient received 3 injections of 10 mg and 1 of 5 mg, making a total of 35 mg/day in 2 days. On the second day, particularly, he received a set of injections with dosages of IM haloperidol 10 mg and diphenhydramine 50 mg in the early a.m. and another set at night. Eighteen minutes after the night injection, a staff member checking the room reported that the patient displayed leg movements and 15 minutes later (33 minutes after the haloperidol injection) he was found unresponsive. Cardiopulmonary resuscitation (CPR) was started but the automated external defibrillator (AED) advised no shock. The emergency medical service (EMS) arrived, pronouncing the patient dead 1 hour and 13 minutes after the last haloperidol IM injection of 10 mg.

#### 3.1.3. Autopsy

The autopsy showed no obvious anomalies in the heart or other organs that would explain the patient's sudden death. In macroscopic and microscopic examination, the coronary arteries and myocardium were essentially normal.

#### 3.1.4. ADR Scales

Using the Liverpool ADR Causality Assessment Tool [30], haloperidol-induced sudden death after 4 haloperidol IM injections with an accumulated dose of 35 mg in 2 days was deemed probable. The patient had not taken any other antipsychotic for weeks. Similarly, based on the Naranjo scale [31], a score of positive 6 (+6) was determined (scoring +1 on items 1, 5, 8, and 10 and +2 on item 2), consistent with a probable ADR. All authors agreed with the scores on both ADR scales. As the patient had no signs of myocardial ischemia or pulmonary thromboembolism, it appears to the authors that haloperidol had to cause the sudden unexpected death by TdP after the accumulation of high serum haloperidol concentrations, particularly with the fourth and last IM injection. The possible contribution of diphenhydramine to this case and to the second case is discussed in detail in the Discussion (see Section 4.5).

As far as we can tell, the literature typically describes TdP as short-duration phenomena (usually seconds) with two outcomes: (1) death due to cardiac arrest or (2) disappearance but with substantial risk of recurrence [34, 35]. Therefore, it may not be not surprising that the AED did not recommend an electrical shock which may suggest that TdP was not present at the time CPR was initiated in this patient. We do not know how long the patient had been unconscious or in cardiac arrest; we only know that his unconsciousness was identified 33 minutes after the last haloperidol IM injection.

### 3.2. Case  2

#### 3.2.1. Prior History

This patient was a 42-year-old African-American female who had given birth to one son. She had been diagnosed with chronic schizophrenia since age 18 and had 8 prior admissions to Kentucky state psychiatric facilities. The court had previously declared her legally incompetent and a sister was appointed to make decisions for her. However, in Kentucky, guardians cannot involuntarily admit patients to psychiatric hospitals; a court needs to approve these admissions.

#### 3.2.2. Last Admission

The patient's 9th admission to a state psychiatric facility was precipitated by the worsening of her psychotic behavior. She was taken to the emergency room in a university hospital with short-term beds under an involuntary order allowing a 72-hour examination before deciding whether or not to ask a court for an involuntary psychiatric admission. After the filing of court documents for an involuntary admission, she was transferred to a psychiatric state hospital. The admission lasted a total of 7 days until her death. She stayed 4 days in the emergency room and 3 days at the psychiatric hospital.

The psychiatric diagnosis was chronic paranoid schizophrenia. Her medical diagnoses included obesity, diabetes mellitus type 2, hypertension, hyperlipidemia, asthma, gastroesophageal reflux disease (GERD), seasonal allergy, and valproate-induced alopecia. Her weight was 131.5 kg (290 pounds) and her body mass index 45.5. At the emergency room, the sister reported that the patient was taking divalproex sodium 1000 mg/day (500 mg twice a day) and paliperidone palmitate injections. The sister explained that divalproex sodium had recently been changed to topiramate due to alopecia, but this change had not yet been implemented; moreover, the patient had been noncompliant with the medication at home. The community mental health center providing outpatient treatment reported that the patient had received paliperidone palmitate 156 mg every month for 2 years with the last injection 16 days before the admission. Her medical medications were, for diabetes, insulin glargine 10 units/day and metformin 2000 mg/day (1000 mg twice a day); for hypertension, lisinopril 5 mg/day; and for allergies, loratadine 10 mg/day.

In the emergency room, she was paranoid, tried to hit the admitting psychiatrist, and showed bizarre behaviors, disrobing and defecating on the bathroom floor. A 5 mg haloperidol IM injection was given and oral psychiatric medication was started, including oral haloperidol, 10 mg/day (5 mg twice a day), and divalproex sodium, 1000 mg/day (500 mg twice a day). The admitting psychiatrist also wrote an “as-needed” oral order for agitation and/or aggression combining 5 mg haloperidol, 2 mg lorazepam, and 25 mg diphenhydramine. These three combined oral drugs (or a single oral drug) could be administered at the discretion of the nurses but with a frequency of not more than every 6 hours. For her medical problems, a diet for diabetic patients was started and aspirin 81 mg/day was added to her prior medical medications (insulin glargine, 10 units/day; metformin, 2000 mg/day; lisinopril, 5 mg/day; and loratadine, 10 mg/day). An EKG on admission (first day at the emergency room after a haloperidol 5 mg IM injection) revealed QT of 336 ms, QTc of 457 ms, and sinus tachycardia (frequency of 111 beats per minute). The routine blood laboratory analyses on the day of admission showed normal electrolytes but presented several abnormalities including glucose of 472 mg/dL (normal range 65–110), white blood cell count of 11,700 leukocytes/mm^3^ (normal range 4,100–10,800), neutrophil count of 7,900 neutrophil/mm^3^ (normal range 1,700–6,000), platelet count of 503,000 platelets/mm^3^ (normal range 140,000–370,000), and glycosylated hemoglobin at 10.4% (normal range 4.8–5.9). Her valproic acid level was undetectable (<3 *μ*g/mL). Her urine showed high glucose, moderate ketones, a few epithelial cells and traces of bacteria. In summary, the laboratory abnormalities suggested that she had not been compliant with her valproate or her antidiabetic medication. Three days later, some of the abnormal levels were reduced: glucose was 330 mg/dL, white blood cell count of 11,740 leukocytes/mm^3^, neutrophil count of 4,500 neutrophil/mm^3^ (a normal value), and platelet count of 497,000 platelets/mm^3^. A lipid profile showed normal values for triglycerides, 133 mg/dL (range 35–150), for cholesterol, 175 mg/dL (range 105–200), and for high density lipoproteins, 37 mg/dL but an elevated low density lipoprotein of 112 mg/dL (range 0–99).

At the psychiatric hospital, the patient was very agitated, psychotic, paranoid, and uncooperative. She would try to walk into other patients' rooms, including male rooms, and touch staff and other patients. These behaviors could not be controlled by having one staff member closely watching her, since she did not listen to redirection. In the state psychiatric hospital over the 3-day period, the antipsychotic dosage progressively accumulated in addition to the divalproex sodium, 1000 mg/day. On day 3 before her death, she received 10 mg of scheduled oral haloperidol and an extra IM injection of 10 mg of haloperidol (with 2 mg IM lorazepam). On day 2 before her death, she received 10 mg of scheduled oral haloperidol. In the early morning (5 AM), she received an extra oral combination of 5 mg of haloperidol, 2 mg of lorazepam, and 25 mg of diphenhydramine. At night, she was agitated and trying to get into the rooms of other patients and at 11 PM she again received extra oral doses of 5 mg of haloperidol, 2 mg of lorazepam, and 25 mg of diphenhydramine. Forty-five minutes later, as she still could not sleep, she was given 50 mg of oral hydroxyzine pamoate. Shortly thereafter, on the third day of admission, in the early AM, the psychiatrist on call was contacted because the patient continued exhibiting the same behaviors and could not receive another haloperidol dose until 6 hours had passed; the psychiatrist ordered the combination of 2 IM injections, 20 mg IM of ziprasidone and 25 mg diphenhydramine at 1:19 AM (2 hours and 19 minutes after the last extra dose of oral haloperidol). Finally, at 2:20 AM she was described as resting quietly in her room. At 7:15 AM, when staff asked if the patient wanted breakfast, she opened her eyes and responded with a gesture that she did not want it and went back to sleep. At 9:00 AM her breathing sounded abnormal to a staff member, who called for a nurse; the nurse found the patient unresponsive with no radial pulse and a faint carotid pulse. This was 7 hours and 41 minutes after the 20 mg ziprasidone IM injection and approximately 10 hours after the last extra dose of oral haloperidol. CPR was started but the AED advised no shock. The EMS arrived and found her in asystole, so at 9:40 AM the patient was declared dead.

#### 3.2.3. Autopsy

The autopsy revealed no obvious anomalies in the heart or other organs that would explain the patient's sudden death. It is important to stress that in spite of her metabolic syndrome, there were no signs of atherosclerosis in the coronary arteries nor any sign of myocardial ischemia in macroscopic or microscopic examinations. There was no evidence of pulmonary thromboembolism. There were some minor abnormalities including mild atherosclerosis at the thoracoabdominal aorta, liver changes compatible with a fatty liver, and a small thyroid mass that microscopic examination demonstrated to be a follicular neoplasm. Postmortem blood toxicology for diphenhydramine was 172 ng/mL (reference range: 30–300 ng/mL) and for hydroxyzine was 35.4 ng/mL (reference range: 22–80 ng/mL).

#### 3.2.4. ADR Scales

Using the Liverpool ADR Causality Assessment Tool [30], antipsychotic-induced sudden death was deemed probable. The last ziprasidone IM injection of 20 mg was considered crucial, because it was given in addition to (1) three extra haloperidol doses (approximately 2 hrs before the ziprasidone injection, 5 mg oral haloperidol; approximately 21 hours earlier, 5 mg oral haloperidol; and 2 days before, one 10 mg IM haloperidol), (2) 10 mg/day of scheduled oral haloperidol for 6 days prior to death, and (3) a long-acting paliperidone injection of 156 mg 18 days prior to death. Similarly, on the Naranjo scale [31], a score of +6 was determined (scoring +1 on items 1, 5, 8, and 10 and +2 on item 2), consistent with a probable ADR. All the authors agreed with the scores on both ADR scales. As the patient had no signs of myocardial ischemia or pulmonary thromboembolism, it appears that the combination of antipsychotics, including the addition of IM ziprasidone on top of IM haloperidol, oral haloperidol, and long-acting paliperidone caused the sudden, unexpected death by TdP. The ziprasidone peak from the IM injection probably combined with the slower peak of the extra oral haloperidol administered 2 hours earlier causing the TdP early the following morning. Oral absorption is much slower than IM absorption and the oral haloperidol absorption may have been further delayed by the combination of an antimuscarinic drug, diphenhydramine.

The possible contribution of diphenhydramine to this case and to the first case is discussed in detail in the Discussion (see Section 4.5).

## 4. Discussion

### 4.1. Limitations of the Patient Sample

To identify these 2 sudden deaths, possibly explained by TdP associated with antipsychotics, we started with 95 deaths reviewed retrospectively using the judgment of a committee composed of psychiatrists, nurses, and pharmacists who have expertise in the subject. During the committee's 13 years of reviewing deaths, judgments regarding the medical cause of death were made after discussion by 2 or 3 psychiatrists who were present at the meetings. The senior author was involved in all reviews of all patients and attended all meetings. We know that the weakest part of our reviews is the limited number of autopsies, 17% (16/95). For years, we have encouraged their use, but we have not been too successful in increasing their numbers, particularly due to cost. We were fortunate to have autopsies of these 2 cases that were highly compatible with TdP. Even with all the limitations of our retrospective reviews, we have accumulated substantial experience with >500 deaths in our state facilities and >350 deaths of psychiatric patients in the community.

### 4.2. Limitations of the Case Reports

It is difficult to make causal connections in case reports. Both deaths were sudden and unexpected, with no signs of cardiac ischemia in the autopsies. They were, however, associated with the accumulation of high serum antipsychotic concentrations after IM injections, compatible with probable ADRs, according to the scales. There are other rare causes of sudden death (e.g., genetic long QT syndrome, Brugada syndrome, or catecholaminergic polymorphic ventricular tachycardia) that cannot be completely ruled out [36], but the proximity of the high doses of IM antipsychotics and the lack of known family histories of sudden deaths in both patients suggested that these two sudden deaths were most likely explained by antipsychotic-induced TdP. Electrolyte abnormalities may contribute to TdP. The first patient refused laboratory testing but was a healthy young male and the second patient did not have any electrolyte abnormality, although magnesium is not routinely measured in our hospitals. In summary, a reasonable interpretation is that these two sudden deaths were unfortunate cases of TdP secondary to the accumulation of high serum peaks of antipsychotics after the last IM injection.

Sinus bradycardia is a risk factor for TdP [16]. The first patient had an EKG with sinus bradycardia 2 years before his death. The EKG described a frequency of 58 beats per minute which appears to be mild bradycardia to us. We do not know if he had sinus bradycardia at any time during the admission leading to his death since the patient refused EKG and vital signs except at the time of admission when he was tachycardic. It is possible that a sinus bradycardia may have contributed to the development of TdP in the first case but we have no way to prove or disprove it.

### 4.3. African-American Race

Knowing that less than 10% of our patients are African-American, we were surprised that both of these deceased patients were African-American. If we assume a frequency of 10% among our patients at psychiatric hospitals in Kentucky, the chance probability that one deceased patient is African-American is 0.10. The chance probability that both are African-Americans is very low: 0.01 (obtained by multiplying 0.10 by 0.10). This is lower than the typical set value of *p* < 0.05 and suggests that the association of African-American race with 100% (2/2) of the antipsychotic-induced sudden deaths may not be explained by chance. We also formally tested the hypothesis of the association between African-American race and TdP by using as a control the prevalence of African-Americans in sudden deaths not compatible with TdP. A 2-sided Fisher exact test provided a significant difference (*p* = 0.016) when comparing 100% (2/2) of African-Americans in sudden deaths compatible with TdP versus 7.5% (2/26) of African-Americans in sudden deaths that were not considered to be explained by TdP and in which the race was known.

In spite of the very small sample size of 2 deaths, both tests of significance were compatible with the possibility that African-American patients may have more risk of death from antipsychotic-induced TdP in our sample. Let us for the moment assume that, as a matter of fact, African-Americans may have a higher risk of dying in our psychiatric hospitals by antipsychotic-induced TdP. Is there any pharmacological mechanism that may explain this association? Yes, there is one; it is the possibility that African-Americans are prone to a higher prevalence of being poor metabolizers (PMs) of haloperidol. Haloperidol metabolism has never been systematically studied to the extent that new drugs are currently studied before they are introduced in the US market. This is important because some old antiepileptics were found to have completely unexpected pharmacokinetic properties when intensively studied prior to introduction in the US market in the 2000s [37]. Psychiatric textbooks and review articles [38] usually indicate that CYP2D6 PMs have decreased ability to eliminate haloperidol. A study of 31 subjects taking haloperidol as a baseline for a clozapine RCT provided an unexpected result [39]. The persistence of serum haloperidol concentrations after haloperidol discontinuation was used to calculate haloperidol elimination half-life in each patient as an approximated marker of haloperidol metabolism. After eliminating patients who had taken haloperidol decanoate, there were a total of 26 patients, of which 16 patients had a half-life <3 days, suggestive of relatively normal haloperidol metabolism, and 10 patients had a half-life ≥3 days, compatible with being haloperidol PMs. Not surprisingly, the only 2 CYP2D6 PMs were in the group with a half-life ≥3 days, suggesting that, in effect, not having CYP2D6 impairs one's ability to eliminate haloperidol. Unexpectedly, the only significant difference (with a *p* = 0.014) between the 16 haloperidol normal metabolizers and the 10 haloperidol PMs was that all 4 African-Americans were haloperidol PMs and had half-lives ≥3 days (as a matter of fact, all 4 patients had half-lives >7 days) [39]. As haloperidol is at least partly metabolized by glucuronidation and some of the glucuronidation enzymes have lower activity in African-Americans [40], it is possible to hypothesize that some African-Americans may have lower ability to eliminate haloperidol due to decreased activity in haloperidol glucuronidation. Recently, an in vitro study of haloperidol metabolism using human hepatocytes has suggested that the uridine diphosphate glucuronosyltransferase (UGT) 2B7 (UGT2B7) may be important in the metabolism of haloperidol [41]. Although there is substantial variation of UGT2B7 alleles worldwide [42], the clinical relevance of these genetic variations for people with African ancestry is not known [43]. In summary, there is very limited information on haloperidol metabolism, but it is possible that some African-Americans may have impaired ability to eliminate haloperidol. If our two patients had impaired ability to eliminate haloperidol, that impairment may have contributed to higher serum haloperidol concentrations, leading to greater risk for TdP. As the haloperidol serum concentration level increases, the blockade of cardiac potassium channels would likewise increase.

### 4.4. Accumulation of One or Several Antipsychotic Formulations

We think that, in these two patients, the last antipsychotic IM injection served as “the straw that broke the camel's back” by providing an even higher peak in serum antipsychotic concentration which contributed to further blockade of cardiac potassium channels encoded by HERG reaching a very high level, leading to TdP. In the first patient, this was achieved after 4 haloperidol IM injections with an accumulated dosage of 25 mg in 2 days before the fourth and last injection of 10 mg, making a total of 35 mg. In the second patient, it was achieved after the last ziprasidone IM injection of 20 mg in addition to (1) three extra haloperidol doses (approximately 2 hours before the ziprasidone injection, 5 mg oral haloperidol; approximately 21 hours earlier, 5 mg oral haloperidol; and 2 days before, one 10 mg IM haloperidol), (2) 10 mg/day of scheduled oral haloperidol for 6 days prior to death, and (3) a long-acting paliperidone injection of 156 mg 18 days prior to death. The second patient received 5 mg oral haloperidol at 11 PM; therefore, the nurse could not administer another as-needed haloperidol dose until 5 AM the next morning and called the psychiatrist on call. In retrospect, we think that the on-call psychiatrist who responded to the nurse and prescribed 20 mg IM of ziprasidone at 1:19 AM on top of the prior extra dose of oral haloperidol made a huge mistake. Both ziprasidone and haloperidol appear to be potent blockers of cardiac potassium channels in clinical doses [11]. Therefore, in this second case, the effects of ziprasidone blocking the cardiac potassium channels encoded by HERG were added to the blocking effects of the prior haloperidol dose. In retrospect, it would have been better to control the agitation of this patient only with lorazepam, which has no relevant effects on the QTc interval [29].

### 4.5. Comedications in Addition to Antipsychotics

The only comedication received by the first patient was IM diphenhydramine. The second patient received, besides IM diphenhydramine, several medications (valproate, insulin, metformin, lisinopril, lorazepam, and hydroxyzine), which have not been associated with TdP or relevant QTc prolongation.

Diphenhydramine has been associated with (1) TdP, occasionally during voluntary overdoses [44–46]; (2) a strong signal for TdP in the databases from drug regulatory agencies [47]; (3) QTc prolongations during voluntary overdoses [48–52], and (4) QTc prolongations in EKGs completed at the emergency room when prescribed by itself or with other drugs that prolong the QTc interval [53]. However, some of these diphenhydramine overdose studies with EKG data, including 126 cases of overdoses with no TdP case [48] and 1 case monitored for several days [50], suggested that diphenhydramine causes tachycardia and this has a major effect on protecting from TdP in situations of QTc prolongation.

The accumulated diphenhydramine dosage was 200 mg IM during the last 3 days of Case  1 and 75 mg during the last 2 days of Case  2 (25 mg IM 2 days prior to death and 50 mg oral on the day before death). We doubt that these doses of diphenhydramine had any relevance in the sudden deaths of these two patients because (1) TdP induced by diphenhydramine overdoses appears to require huge quantities measured in grams [44, 45], (2) diphenhydramine-induced tachycardia may protect from TdP in situations of QTc prolongation [48, 50], and (3) some animal models of clinical relevance in humans for drugs that prolong QTc cannot detect any effect for diphenhydramine doses trying to replicate clinical dosing [54].

### 4.6. Reflections on the History of Antipsychotic-Induced TdP

The next section discusses a practice guideline limiting high doses of haloperidol and/or ziprasidone IM injections in the hope of preventing the repetition of similar TdP cases in our Kentucky facilities. Ideally, practice guidelines should be based on principles following the hierarchical thinking proposed by evidence-based medicine, which gives higher value to RCTs. In the real world, those researchers insisting on the evidence-based medicine (EBM) approach are not aware that evidence related to ADRs is seriously distorted by the effects of pharmaceutical companies to cover the ADRs resulting from their drugs and their lack of attention to ADRs during studies undertaken for market approval [55]. Moreover, RCTs focus on mean effects, but when a very rare ADR, such as TdP, is studied, mean values are irrelevant and outliers are important [56]. Therefore, some authors can dismiss the risk of haloperidol-induced TdP because it is mainly based on case reports and not RCT data [57]. How unwise is it to ask clinicians to wait for RCT data on haloperidol-induced TdP? It is very unwise; a randomized study of >18,000 patients followed for 1 year had no statistical power to detect ziprasidone-induced TdP [18]. Haloperidol is off-patent; therefore, nobody is going to finance a large RCT of 10,000s of patients for several years to detect statistical differences between haloperidol and other drugs in causing TdP. Imagine that the thioridazine marketer had paid attention to Ban [1] in the late 1960s, performed more studies, and withdrawn thioridazine from the market instead of waiting until 2006. The outcome of that decision, based on “limited” case reports would probably have led to saving hundreds or thousands of lives worldwide during the last 30 years. Psychiatrists would have prescribed other antipsychotics with less risk for TdP rather instead of prescribing thioridazine, which may be the oral antipsychotic that most frequently causes sudden death [58]. Similarly, it would be a mistake to ignore the risk of TdP associated with high doses of haloperidol IM, suggested by our 2 case reports.

### 4.7. Practice Guidelines

Both deaths were considered possibly preventable if the prescribing psychiatrist had better understood the risk of a standing as-needed order for agitation using IM antipsychotics with potential to cause TdP. This type of order is written for all our patients but is rarely used by nurses. If they are used in a patient, IM antipsychotic injections are rarely repeated within a few hours. In the first case, the psychiatrist allowed a high dosage of 10 mg haloperidol IM every 12 hours, which is rarely prescribed in our hospitals (most psychiatrists prescribe 5 mg every 6 hours at most). The psychiatrist thought it was safe because the same order had been written for the same patient at the same hospital 3 years before, but 3 years before it was scarcely used. In the second case, the as-needed order of 5 mg every 6 hours was written as administered by the oral route, but when it was not enough to control the agitation of the patient, the nurses called the on-call psychiatrist who circumvented the limit of a haloperidol dose every 6 hours by adding 20 mg IM ziprasidone in close proximity to the last oral dose of haloperidol.

The medical literature has addressed the risk of TdP during IV haloperidol treatment, but the psychiatric literature including practice guidelines [59] has paid little attention to the risk of TdP after IM haloperidol and/or IM ziprasidone. Therefore, a practice guideline was developed for IM antipsychotics to limit dosing for haloperidol and/or ziprasidone IM in order to prevent future TdP cases and sudden deaths in our public psychiatric hospitals in Kentucky.

The practice guideline that is shown in the Appendix was based on (1) a comprehensive review of the literature on the use of IM antipsychotics to treat agitation, including review articles [60–74] and prior practice guidelines [59, 75–79]; (2) learning from the mistakes in these two cases; and (3) a review of the pharmacokinetic and pharmacodynamic mechanisms behind the DDIs contributing to antipsychotic-induced TdP [14, 80, 81].

## 5. Conclusion

The medical literature has addressed the risk of TdP during IV haloperidol treatment but the psychiatric literature has paid little attention to TdP risk after IM haloperidol and/or IM ziprasidone [59]. In this paper, we presented two cases of sudden death after IM injections of antipsychotics, which were considered to be probable ADRs. The autopsies described lack of heart pathology and were highly compatible with the possibility of TdP in the absence of risk factors other than the accumulation of antipsychotics with an even higher serum peak after a last injection led to death within a few hours. It is possible that these cases were explained by a combination of high serum concentrations of antipsychotics with particular individual vulnerability. Some studies suggest that rare genetic variations at potassium channels may contribute to predisposition to drug-induced TdP [82].

The first case describes a 27-year-old African-American male with schizophrenia but no medical issues. His death occurred in the early morning of the third day and was probably caused by repeated IM haloperidol injections of 10 mg (totaling 35 mg in 2 days). The second case involves a 42-year-old African-American female with a metabolic syndrome. Her death was probably caused by a last ziprasidone IM injection of 20 mg in addition to (1) three extra haloperidol doses (approximately 2 hours before the ziprasidone injection, 5 mg oral haloperidol; approximately 21 hours earlier, 5 mg oral haloperidol; and 2 days prior, one 10 mg IM haloperidol), (2) 10 mg/day of scheduled oral haloperidol for 6 days prior to death, and (3) a long-acting paliperidone injection of 156 mg 18 days prior to death. The association of TdP deaths with African-American race in spite of the small sample size appears to suggest a significant overrepresentation when compared with our patients at Kentucky public psychiatric hospitals. As a matter of fact, patients with poor capacity to metabolize haloperidol might be overrepresented among African-Americans. The study of haloperidol metabolism through glucuronidation in African-Americans is urgently recommended since the percentage of impaired individuals may be overrepresented in African-American individuals. If it is demonstrated that African-Americans have lower ability to metabolize haloperidol, this would indicate the need to consider race and possibly genetics, in the dosing of IM antipsychotics for agitation and for the need to include these differences in the practice guidelines for agitation.

## Appendix

Proposed practice guidelines for dosing parenteral antipsychotics in order to avoid TdP during the control of agitation at psychiatric hospitals are as follows.

*IV Haloperidol*. The use of IV haloperidol is not recommended and it cannot be used unless continuous EKG monitoring is available.

*IM Chlorpromazine*. The use of IM chlorpromazine is not recommended because of the lack of studies and very limited number of RCTs [60] and the potential for oral chlorpromazine to cause TdP and orthostatic hypotension [61]. IM chlorpromazine cannot be used unless there is (1) an absence of any of the risk factors for TdP (see below) and (2) a baseline EKG that shows QTc < 450 ms on the same medication that the patient is currently taking.

*Combining IM Haloperidol and IM Ziprasidone*. Orders for IM haloperidol and IM ziprasidone cannot be combined in the same patient. If a patient has received IM haloperidol, no IM ziprasidone can be administered for 24 hours and if a patient has received IM ziprasidone, no IM haloperidol can be administered for 24 hours.

*IM Haloperidol*

(1) IM Haloperidol cannot be used in any patient with risk factors for TdP (see below).
(2) Standard agitation as-needed orders for IM haloperidol doses ≥10 mg cannot be written unless there is a baseline EKG that shows QTc < 450 ms on the same medication that the patient is currently taking. See below for an exception for patients on potent inducers.
(3) Standard agitation as-needed orders for IM haloperidol doses of 5 mg should not recommend administration with a frequency <12 hours unless there is a baseline EKG that shows QTc < 450 ms on the same medication that the patient is currently taking. The combination of IM haloperidol with IM benzodiazepines and/or IM antihistaminic/anticholinergics has not been systematically studied but is frequently used by our clinicians. To decrease the total number of haloperidol doses and reduce the potential for sedation, clinicians are encouraged to combine haloperidol IM injections of 5 mg with simultaneous administration of a benzodiazepine IM injection (such as IM lorazepam) and/or IM anticholinergic/antihistamine injection (such as IM diphenhydramine). As these latter medications do not appear to be associated with risk for QTc prolongation, they can be administered at frequencies of less than 12 hours.
(4) If there is no baseline EKG while on current medications, it is OK to write standard orders for agitation for haloperidol 5 mg every 12 hours while using other IM drugs with less potential for QTc prolongation more frequently (e.g., for 1 mg of lorazepam every 6 hours and/or for 25 mg of diphenhydramine every 6 hours).
(5) Fluoxetine is at least a mild inhibitor of haloperidol metabolism [81]. In patients taking fluoxetine or who have taken it in the last month (norfluoxetine stays in the body for weeks after discontinuation), clinicians need to be aware that the doses of haloperidol IM that they write may produce higher effects. The limited available published information [81] suggests that adding fluoxetine is equivalent to increasing serum haloperidol concentrations by 33% after 12 weeks on fluoxetine. This is equivalent to multiplying the effects of the haloperidol IM dose by a factor of 1.33 and to compensate, this will require multiplying IM haloperidol doses by 0.75 (calculated by dividing 1/1.33 = 0.75). There are no haloperidol studies of the effects of fluoxetine over several months and at steady state for inhibition of haloperidol metabolism; therefore, it is possible that patients taking fluoxetine may require even lower IM haloperidol doses. It may be safer to avoid IM haloperidol in patients taking fluoxetine since fluoxetine also prolongs QTc.

*IM Ziprasidone*

1. IM ziprasidone cannot be used in any patient with risk factors for TdP (see below).
2. The prescribing information recommends maximum total daily doses of ≤40 mg/day and intervals for doses of ≥2 hours for 10 mg and ≥4 hours for 20 mg injections.
3. Standard agitation as-needed orders for IM ziprasidone of 20 mg should not recommend administration with a frequency of <12 hours unless there is a baseline EKG that shows QTc < 450 ms on the same medication that the patient is currently taking.

*IM Olanzapine*

1. IM olanzapine appears to have less risk for QTc prolongation than IM haloperidol and/or IM ziprasidone. If there is a need for IM antipsychotics in a patient with risk factors for TdP, olanzapine IM is a better choice than haloperidol or ziprasidone IM [65].
2. The olanzapine prescribing information recommends maximum total daily doses of ≤30 mg/day and an interval for 10 mg doses of ≥2 hours after the initial injection and ≥4 hours after the second IM injection.
3. Although oral olanzapine is not usually linked to orthostatic hypotension, IM olanzapine has been associated with syncope. The prescribing information recommends doses ≤5 mg in geriatric patients (≥65 years) and doses of 2.5 mg in patients with risk for orthostatic hypotension.
4. IM olanzapine is a fairly sedating drug and there is no need to combine it with benzodiazepines. As a matter of fact, it should not be combined with IM benzodiazepines since this combination has occasionally been associated with hypotension [71] and even rarely with death [67]. The decision to add an IM olanzapine injection within 24 hours of a benzodiazepine IM injection would require chart documentation for the reason and a written order to closely monitor the patient for orthostatic hypotension and/or respiratory depression to avoid any risk, since we have very limited data about the safety of this combination, which is discouraged by olanzapine's marketer [63]. Obviously, if the patient is lying in bed, there is no need to monitor for orthostatic changes unless the patient needs to get up.
5. If the patient is taking fluvoxamine, IM olanzapine should be used in lower doses due to the inhibition of olanzapine metabolism. The clinician should consider using half the dosage he/she typically uses [14].

*IM Aripiprazole*

1. IM aripiprazole appears to have less risk for QTc prolongation than IM haloperidol and/or IM ziprasidone.
2. The aripiprazole prescribing information recommends (a) maximum total daily doses of ≤30 mg/day, (b) a dose of 9.75 mg (range 5.25–15 mg), and (c) and an interval between injections ≥2 hours.
3. If the patient is taking powerful CY3PA4 inhibitors or CYP2D6 inhibitors (including paroxetine and fluoxetine), halving the doses is recommended. Duloxetine and bupropion are moderate CYP2D6 inhibitors; therefore, using half the dosage used for typical patients appears to be a good idea [14].
4. There is limited information comparing IM aripiprazole with other IM second-generation antipsychotics, but the available information suggests that IM aripiprazole may be less effective for controlling agitation than IM olanzapine or IM ziprasidone [61, 62].

*Patients with Risk for TdP*. Having any of the following is considered a risk factor for TdP: (1) history of sudden death in the family, (2) personal history of syncope, (3) active or residual serious heart disease, (4) geriatric age (≥65 years), (5) bradycardia (<60 beats per minute), (6) hypokalemia, (7) hypomagnesemia, or (7) the use of nonpsychiatric drugs with risk for QTc prolongation including antiarrhythmics (amiodarone dofetilide, quinidine, procainamide, and sotalol), some antibiotics (e.g., gatifloxacin and moxifloxacin), or others (e.g., arsenic trioxide, mefloquine, methadone, pentamidine, and tacrolimus).

Some of these factors can be easily reversed; therefore, correcting electrolyte abnormalities or discontinuing drugs associated with QTc prolongation should be considered before adding orders for IM antipsychotics. IM benzodiazepines do not appear to prolong QTc interval; therefore, it appears safe to use them in patients with risk for TdP [67].

*Patients on Scheduled Antipsychotics or Antidepressants with Risk for TdP.* Some oral antipsychotics (including amisulpride, haloperidol, iloperidone, phenothiazines, pimozide, or ziprasidone), haloperidol decanoate, fluphenazine decanoate, and antidepressants (including SSRIs and TCAs) have been definitively associated with clinically relevant potential to prolong QTc; therefore, it is better to avoid adding IM haloperidol and IM ziprasidone for agitation in a patient taking any of these drugs. IM benzodiazepines or IM olanzapine should be considered for as-needed orders for agitation in these patients. If the clinician considers the riskier path of adding IM haloperidol or IM ziprasidone to these psychiatric medications, he/she needs to consider using lower doses and provide written justification.

*Patients on Inducers*. If the patient is on a potent inducer (e.g., rifampin or some antiepileptic drugs: carbamazepine, phenytoin, or phenobarbital), he/she may metabolize some antipsychotics such as aripiprazole, haloperidol, or olanzapine very fast [80] and may need higher doses of these IM antipsychotics beyond what is typically recommended. If the clinician needs to write higher doses of any of these IM antipsychotics, he/she should consult the pharmacy first before writing an order for unusually high doses of any IM antipsychotics. Potent inducers appear to have small effects on ziprasidone metabolism [80]; therefore, although this has not been studied well, patients taking potent inducers may respond to normal doses of IM ziprasidone. Normal doses of ziprasidone IM may be a rational choice for patients taking potent inducers.

*Patients with Alcohol Intake/Intoxication*. IM antipsychotics should be judiciously used to manage agitation in patients with recent alcohol intake or alcohol intoxication. IM olanzapine [71, 72] and IM ziprasidone with or without benzodiazepines [70] have been associated with decreases in oxygen saturation.

*Individualized Treatment*. This set of guidelines for IM antipsychotics was developed to discourage the use of high doses of IM haloperidol, IM ziprasidone, or the combination of IM haloperidol with IM ziprasidone for standard agitation orders for all patients. It does not appear safe to continue to use nurses' judgment to decide the safe frequency of this type of order when this appears to have led to two sudden deaths in our psychiatric hospitals. In emergency situations, a clinician can overrule the dosing limits in this guideline by using a one-time order prescribing high doses as long as he/she understands the risk and has appropriate safeguards in place (see the IM olanzapine and IM benzodiazepine combinations) and documents them in the chart. Once the emergency situation is resolved, the clinician should be ready to have his/her actions reviewed by the hospital pharmacy that may encourage or discourage similar actions in the future based on the peculiarities of the individual case.

## Abbreviations

EKG: Electrocardiogram
IM: Intramuscular
IV: Intravenous
ms: Milliseconds
SSRI: Selective serotonin reuptake inhibitor
TCA: Tricyclic antidepressant
TdP: Torsades de pointes.

## References

[1] Shorter E. The QT interval and the Mellaril story: a cautionary tale. http://inhn.org/controversies/edward-shorter-the-q-t-interval-and-the-mellaril-story-a-cautionary-tale.html.

[2] Kelly H. G., Fay J. E., Laverty S. G. (1963). Thioridazine hydrochloride (Mellaril): its effect on the electrocardiogram and a report of two fatalities with electrocardiographic abnormalities. *Canadian Medical Association journal*.

[3] Ban T. A., Jean A. St. (1964). The effect of phenothiazines on the electrocardiogram. *Canadian Medical Association Journal*.

[4] Desautels S., Filteau C., Jean A. St. (1964). Ventricular tachycardia associated with administration of thioridazine hydrochloride (Mellaril). *Journal of the Canadian Medical Association*.

[5] Branchey M. H., Lee J. H., Amin R., Simpson G. M. (1978). High- and low-potency neuroleptics in elderly psychiatric patients. *Journal of the American Medical Association*.

[6] Issa A. M., Phillips K. A., Van Bebber S. (2007). Drug withdrawals in the United States: a systematic review of the evidence and analysis of trends. *Current Drug Safety*.

[7] Flockhart D. A. (1996). Drug interactions, cardiac toxicity, and terfenadine: from bench to clinic?. *Journal of Clinical Psychopharmacology*.

[8] Muscatello M. R. A., Bruno A., Pandolfo G., Micò U., Settineri S., Zoccali R. (2010). Emerging treatments in the management of schizophrenia—focus on sertindole. *Drug Design, Development and Therapy*.

[9] Rampe D., Murawsky M. K., Grau J., Lewis E. W. (1998). The antipsychotic agent sertindole is a high affinity antagonist of the human cardiac potassium channel HERG. *Journal of Pharmacology and Experimental Therapeutics*.

[10] Shader R. I., Greenblatt D. J. (1998). Potassium, antipsychotic agents, arrhythmias, and sudden death. *Journal of Clinical Psychopharmacology*.

[11] Harrigan E. P., Miceli J. J., Anziano R. (2004). A randomized evaluation of the effects of six antipsychotic agents on QTc, in the absence and presence of metabolic inhibition. *Journal of Clinical Psychopharmacology*.

[12] Glassman A. H., Bigger J. T. (2001). Antipsychotic drugs: prolonged QTc interval, torsade de pointes, and sudden death. *American Journal of Psychiatry*.

[13] Ramos-Ríos R., Arrojo-Romero M., Paz-Silva E. (2010). QTc interval in a sample of long-term schizophrenia inpatients. *Schizophrenia Research*.

[14] Spina E., de Leon J. (2014). Clinically relevant interactions between newer antidepressants and second-generation antipsychotics. *Expert Opinion on Drug Metabolism and Toxicology*.

[15] Heist E. K., Ruskin J. N. (2010). Drug-induced arrhythmia. *Circulation*.

[16] Kannankeril P., Roden D. M., Darbar D. (2010). Drug-induced long QT syndrome. *Pharmacological Reviews*.

[17] Wenzel-Seifert K., Wittmann M., Haen E. (2011). QTc prolongation by psychotropic drugs and the risk of torsade de pointes. *Deutsches Ärzteblatt International*.

[18] Strom B. L., Eng S. M., Faich G. (2011). Comparative mortality associated with ziprasidone and olanzapine in real-world use among 18,154 patients with schizophrenia: The Ziprasidone Observational Study of Cardiac Outcomes (ZODIAC). *The American Journal of Psychiatry*.

[19] Camm A. J., Karayal O. N., Meltzer H. (2012). Ziprasidone and the corrected QT interval: a comprehensive summary of clinical data. *CNS Drugs*.

[20] Poluzzi E., Raschi E., Koci A. (2013). Antipsychotics and torsadogenic risk: signals emerging from the US FDA adverse event reporting system database. *Drug Safety*.

[21] Kongsamut S., Kang J., Chen X.-L., Roehr J., Rampe D. (2002). A comparison of the receptor binding and HERG channel affinities for a series of antipsychotic drugs. *European Journal of Pharmacology*.

[22] Li X. B., Tang Y. L., Zheng W., Wang C., de Leon J. (2014). QT interval prolongation associated with intramuscular ziprasidone in chinese patients: a case report and a comprehensive literature review with meta-analysis. *Case Reports in Psychiatry*.

[23] Metzger E., Friedman R. (1993). Prolongation of the corrected QT and torsades de pointes cardiac arrhythmia associated with intravenous haloperidol in the medically ill. *Journal of Clinical Psychopharmacology*.

[24] Sharma N. D., Rosman H. S., Padhi I. D., Tisdale J. E. (1998). Torsades de pointes associated with intravenous haloperidol in critically ill patients. *The American Journal of Cardiology*.

[25] Hatta K., Takahashi T., Nakamura H. (2001). The association between intravenous haloperidol and prolonged QT interval. *Journal of Clinical Psychopharmacology*.

[26] Hassaballa H. A., Balk R. A. (2003). Torsade de pointes associated with the administration of intravenous haloperidol. *American Journal of Therapeutics*.

[27] Meyer-Massetti C., Cheng C. M., Sharpe B. A., Meier C. R., Guglielmo B. J. (2010). The FDA extended warning for intravenous haloperidol and torsades de pointes: how should institutions respond?. *Journal of Hospital Medicine*.

[28] Jackson T., Ditmanson L., Phibbs B. (1997). Torsade de pointes and low-dose oral haloperidol. *Archives of Internal Medicine*.

[29] Harvey A. T., Flockhart D., Gorski J. C. (2004). Intramuscular haloperidol or lorazepam and QT intervals in schizophrenia. *Journal of Clinical Pharmacology*.

[30] Gallagher R. M., Kirkham J. J., Mason J. R. (2011). Development and inter-rater reliability of the Liverpool adverse drug reaction causality assessment tool. *PLoS ONE*.

[31] Naranjo C. A., Busto U., Sellers E. M. (1981). A method for estimating the probability of adverse drug reactions. *Clinical Pharmacology and Therapeutics*.

[32] de Leon J., Mallory P., Maw L., Susce M. T., Perez-Rodriguez M. M., Baca-Garcia E. (2011). Lack of replication of the association of low serum cholesterol and attempted suicide in another country raises more questions. *Annals of Clinical Psychiatry*.

[33] de Leon J., Susce M. T., Johnson M. (2009). DNA microarray technology in the clinical environment: the Amplichip CYP450 test for CYP2D6 and CYP2C19 genotyping. *CNS Spectrums*.

[34] Tzivoni D., Banai S., Schuger C. (1988). Treatment of torsade de pointes with magnesium sulfate. *Circulation*.

[35] Cleland J. G. F., Krikler D. M. (1992). Torsade de pointes: chaos, sixteen years on?. *British Heart Journal*.

[36] Vyas V., Lambiase P. D. (2013). The investigation of sudden arrhythmic death syndrome (SADS)-the current approach to family screening and the future role of genomics and stem cell technology. *Frontiers in Physiology*.

[37] de Leon J. (2014). False-negative studies may systematically contaminate the literature on the effects of inducers in neuropsychopharmacology—part I: focus on epilepsy. *Journal of Clinical Psychopharmacology*.

[38] Spina E., de Leon J. (2015). Clinical applications of CYP genotyping in psychiatry. *Journal of Neural Transmission*.

[39] de Leon J., Diaz F. J., Wedlund P., Josiassen R. C., Cooper T. B., Simpson G. M. (2004). Haloperidol half-life after chronic dosing. *Journal of Clinical Psychopharmacology*.

[40] de Leon J. (2003). Glucuronidation enzymes, genes and psychiatry. *International Journal of Neuropsychopharmacology*.

[41] Kato Y., Nakajima M., Oda S., Fukami T., Yokoi T. (2012). Human UDP-glucuronosyltransferase isoforms involved in haloperidol glucuronidation and quantitative estimation of their contribution. *Drug Metabolism and Disposition*.

[42] Čolić A., Alessandrini M., Pepper M. S. (2015). Pharmacogenetics of CYP2B6, CYP2A6 and UGT2B7 in HIV treatment in African populations: focus on efavirenz and nevirapine. *Drug Metabolism Reviews*.

[43] Li J., Menard V., Benish R. L. (2012). Worldwide variation in human drug-metabolism enzyme genes *CYP2B6* and *UGT2B7*: implications for HIV/AIDS treatment. *Pharmacogenomics*.

[44] Joshi A. K., Sljapic T., Borghei H., Kowey P. R. (2004). Case of polymorphic ventricular tachycardia in diphenhydramine poisoning. *Journal of Cardiovascular Electrophysiology*.

[45] Husain Z., Hussain K., Nair R., Steinman R. (2010). Diphenhydramine induced QT prolongation and torsade de pointes: an uncommon effect of a common drug. *Cardiology Journal*.

[46] Al-Abri S. A., Woodburn C., Olson K. R., Kearney T. E. (2015). Ventricular dysrhythmias associated with poisoning and drug overdose: a 10-year review of statewide poison control center data from California. *American Journal of Cardiovascular Drugs*.

[47] Poluzzi E., Raschi E., Godman B. (2015). Pro-arrhythmic potential of oral antihistamines (H1): combining adverse event reports with drug utilization data across Europe. *PLoS ONE*.

[48] Zareba W., Moss A. J., Rosero S. Z., Hajj-Ali R., Konecki J., Andrews M. (1997). Electrocardiographic findings in patients with diphenhydramine overdose. *The American Journal of Cardiology*.

[49] Thakur A. C., Aslam A. K., Aslam A. F., Vasavada B. C., Sacchi T. J., Khan I. A. (2005). QT interval prolongation in diphenhydramine toxicity. *International Journal of Cardiology*.

[50] Sype J. W., Khan I. A. (2005). Prolonged QT interval with markedly abnormal ventricular repolarization in diphenhydramine overdose. *International Journal of Cardiology*.

[51] Ramachandran K., Sirop P. (2008). Rare complications of diphenhydramine toxicity. *Connecticut Medicine*.

[52] Chen T.-Y., Yeh Y.-W., Kuo S.-C., Chen C.-Y., Lin T.-P., Chang C.-C. (2014). Diphenhydramine dependence through deep intramuscular injection resulting in myonecrosis and prolonged QT interval. *Journal of Clinical Pharmacy and Therapeutics*.

[53] Tay K.-Y., Ewald M. B., Bourgeois F. T. (2014). Use of QT-prolonging medications in US emergency departments, 1995–2009. *Pharmacoepidemiology and Drug Safety*.

[54] Toyoshima S., Kanno A., Kitayama T. (2005). QT PRODACT: in vivo QT assay in the conscious dog for assessing the potential for QT interval prolongation by human pharmaceuticals. *Journal of Pharmacological Sciences*.

[55] Ioannidis J. P. A. (2009). Adverse events in randomized trials: neglected, restricted, distorted, and silenced. *Archives of Internal Medicine*.

[56] de Leon J. (2012). Evidence-based medicine versus personalized medicine: are they enemies?. *Journal of Clinical Psychopharmacology*.

[57] Howland R. H. (2014). QTc prolongation and haloperidol: just how risky is this drug?. *Psychosomatics*.

[58] Salvo F., Pariente A., Shakir S. (2016). Sudden cardiac and sudden unexpected death related to antipsychotics: a meta-analysis of observational studies. *Clinical Pharmacology and Therapeutics*.

[59] Pacciardi B., Mauri M., Cargioli C. (2013). Issues in the management of acute agitation: how much current guidelines consider safety?. *Frontiers in Psychiatry*.

[60] Ahmed U., Jones H., Adams C. E. (2010). Chlorpromazine for psychosis induced aggression or agitation. *Cochrane Database Systematic Reviews*.

[61] Battaglia J. (2005). Pharmacological management of acute agitation. *Drugs*.

[62] Bosanac P., Hollander Y., Castle D. (2013). The comparative efficacy of intramuscular antipsychotics for the management of acute agitation. *Australasian Psychiatry*.

[63] Caine E. D. (2006). Clinical perspectives on atypical antipsychotics for treatment of agitation. *Journal of Clinical Psychiatry*.

[64] Citrome L. (2007). Comparison of intramuscular ziprasidone, olanzapine, or aripiprazole for agitation: a quantitative review of efficacy and safety. *Journal of Clinical Psychiatry*.

[65] Kishi T., Matsunaga S., Iwata N. (2015). Intramuscular olanzapine for agitated patients: a systematic review and meta-analysis of randomized controlled trials. *Journal of Psychiatric Research*.

[66] Powney M. J., Adams C. E., Jones H. (2012). Haloperidol for psychosis-induced aggression or agitation (rapid tranquillisation). *Cochrane Database of Systematic Reviews*.

[67] Ries R., Sayadipour A. (2014). Management of psychosis and agitation in medical-surgical patients who have or are at risk for prolonged QT interval. *Journal of Psychiatric Practice*.

[68] San L., Arranz B., Escobar R. (2005). Pharmacological management of acutely agitated schizophrenic patients. *Current Pharmaceutical Design*.

[69] Tulloch K. J., Zed P. J. (2004). Intramuscular olanzapine in the management of acute agitation. *Annals of Pharmacotherapy*.

[70] Wilson M. P., Macdonald K., Vilke G. M., Ronquillo L., Feifel D. (2013). Intramuscular ziprasidone: influence of alcohol and benzodiazepines on vital signs in the emergency setting. *Journal of Emergency Medicine*.

[71] Wilson M. P., Chen N., Vilke G. M., Castillo E. M., MacDonald K. S., Minassian A. (2012). Olanzapine in ED patients: differential effects on oxygenation in patients with alcohol intoxication. *American Journal of Emergency Medicine*.

[72] Wilson M. P., MacDonald K., Vilke G. M., Feifel D. (2012). Potential complications of combining intramuscular olanzapine with benzodiazepines in emergency department patients. *Journal of Emergency Medicine*.

[73] Zacher J. L., Roche-Desilets J. (2005). Hypotension secondary to the combination of intramuscular olanzapine and intramuscular lorazepam. *Journal of Clinical Psychiatry*.

[74] Zimbroff D. L. (2008). Pharmacological control of acute agitation: focus on intramuscular preparations. *CNS Drugs*.

[75] Allen M. H., Currier G. W., Carpenter D., Ross R. W., Docherty J. P., Expert Consensus Panel for Behavioral Emergencies 2005 (2005). The expert consensus guideline series. Treatment of behavioral emergencies 2005. *Journal of Psychiatric Practice*.

[76] Marder S. R. (2006). A review of agitation in mental illness: treatment guidelines and current therapies. *Journal of Clinical Psychiatry*.

[77] Canadian Agency for Drugs and Technologies in Health (2015). *Use of Antipsychotics and/or Benzodiazepines as Rapid Tranquilization in In-Patients of Mental Facilities and Emergency Departments: A Review of the Clinical Effectiveness and Guidelines*.

[78] The British Psychological Society and The Royal College of Psychiatrists (2015). *Violence and Aggression: Short-Term Management in Mental Health, Health and Community Settings*.

[79] Tylor D., Paton C., Kapur S. (2012). *The Maudsley Prescribing Guidelines in Psychiatry*.

[80] de Leon J., Santoro V., D'Arrigo C., Spina E. (2012). Interactions between antiepileptics and second-generation antipsychotics. *Expert Opinion on Drug Metabolism and Toxicology*.

[81] Avenoso A., Spina E., Campo G. (1997). Interaction between fluoxetine and haloperidol: pharmacokinetic and clinical implications. *Pharmacological Research*.

[82] Weeke P., Mosley J. D., Hanna D. (2014). Exome sequencing implicates an increased burden of rare potassium channel variants in the risk of drug-induced long QT interval syndrome. *Journal of the American College of Cardiology*.

